# Primary Squamous Cell Carcinoma of the Renal Pelvis: A Rare Complication of Xanthogranulomatous Pyelonephritis

**DOI:** 10.7759/cureus.44750

**Published:** 2023-09-05

**Authors:** Nuno Maia Neves, Francisco S Laranjeira, Susana Coelho, Anabela Raimundo, Alexandra Bayão Horta

**Affiliations:** 1 Internal Medicine Department, Hospital da Luz Lisboa, Lisbon, PRT

**Keywords:** nephrectomy, staghorn calculi, nephrolithiasis, squamous cell carcinoma, xanthogranulomatous, pyelonephritis

## Abstract

Xanthogranulomatous pyelonephritis is a rare disease resulting from chronic inflammation and infection of the renal parenchyma. It usually arises as a consequence of obstructive chronic pyelonephritis. Primary squamous cell carcinoma of the renal pelvis is a distinct pathology, very rare in clinical practice, with a well-established association with xanthogranulomatous pyelonephritis.

The authors present the case of a 57-year-old woman with chronic pyelonephritis containing xanthogranulomatous features. Subsequent workup revealed a concomitant, unsuspected, primary squamous cell carcinoma of the renal pelvis.

With this case, the authors intend to emphasize and reinforce the need to be alert to an uncommon association between two rare diseases due to its diagnostic, therapeutic, and prognostic implications.

## Introduction

Chronic pyelonephritis can, on rare occasions, evolve into xanthogranulomatous pyelonephritis (XPN). Such progression is most notable in settings of urinary obstruction resulting from infected renal stones. In these cases, massive destruction of the involved kidney can be seen, usually due to focal or diffuse replacement of renal parenchyma by granulomatous tissue containing lipid-filled macrophages [1]. The most important differential diagnosis is renal carcinoma since its clinical presentation and imaging appearance can be similar.

Primary squamous cell carcinoma (SCC) of the renal pelvis and ureter are very rare and represent less than 1% of all urinary tract neoplasms [2-4]. These rare malignancies can be associated with renal stone disease [3,4], probably as a consequence of chronic inflammation or infection due to urinary stasis, which can lead to metaplasia of the urothelial epithelium with subsequent carcinomatous transformation. Although the association between XPN and SCC is well-established, the coexistence of both entities at the same time is very rare, with only a few cases reported.

The authors report the case of a 57-year-old woman with concomitant xanthogranulomatous pyelonephritis and primary squamous cell carcinoma of the renal pelvis, whose rapid decline and decease were unexpected.

## Case presentation

A 57-year-old woman, with a history of asymptomatic nephrolithiasis, presented to the emergency department complaining of left flank pain, significant weight loss (> 10%), anorexia, and asthenia in the previous two months. She reported no fever, hematochezia, melena, or a change in bowel habits or urinary symptoms.

On admission, she was hypotensive (92/57 mmHg) with a pulse rate of 93 beats/minute, oxygen saturation of 94% breathing ambient air, and tympanic temperature of 37.8ºC. The left hemithorax was dull to percussion and breath sounds were absent; she presented abdominal tenderness on the left flank. Blood tests revealed normochromic and normocytic anemia, leukocytosis with neutrophilia, thrombocytosis, acute kidney injury, increased lactate dehydrogenase (LDH), and C-reactive protein (CRP) (Table 1).

**Table 1:** Patient’s laboratory workup on admission g/dL - grams per deciliter; mg/dL - milligrams per deciliter; IU/L - international units per liter; LDH - lactate dehydrogenase; CRP - C-reactive protein

Blood tests	Laboratory results	Reference range
Hemoglobin (g/dL)	8.0	12-15.2
Leucocytes (x 10^9^/L)	25.050	4-11
Neutrophils (x 10^9^/L)	22.500	1.8-7.4
Platelets (x 10^9^/L)	799	150-400
Urea (mg/dL)	105	19-49
Serum creatinine (mg/dL)	2.87	< 1
LDH (IU/L)	850	120-246
Total protein (g/dL)	6.0	6-7.8
CRP (mg/dL)	27.36	< 0.6

**Figure 1:**
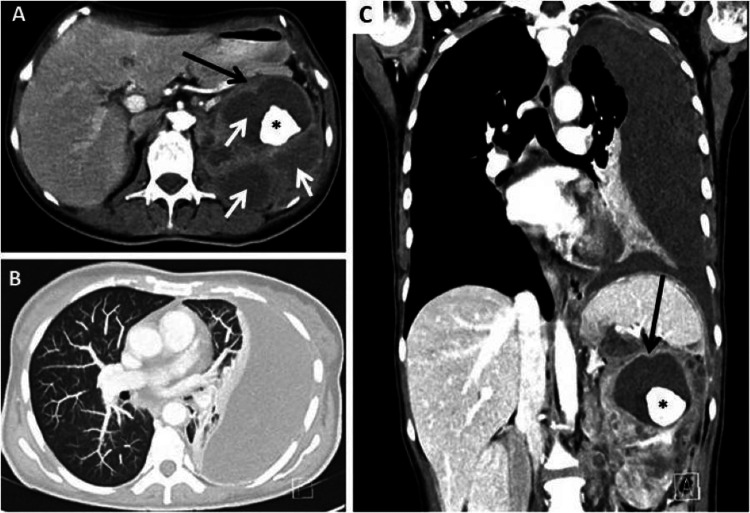
Thoracic and abdominal CT Images show a structural alteration of the left kidney with almost complete substitution of the parenchyma by cystic lesions, with dimensions varying from millimeters to 7 centimeters (Figures 1A, 1C; black arrow), some of these with gross intraluminal calcifications and a staghorn calculus (Figures 1A, 1C; asterisk). Delayed uptake of contrast in the affected kidney was documented reflecting hypofunction of the renal parenchyma. Several hypoattenuating round areas throughout the enlarged kidney resembling the paw print of a bear (bear paw sign) can be seen in Figure 1A (white arrows). In Figures 1B, 1C, a large pleural effusion with passive atelectasis of left inferior lobe can be seen.

**Figure 2:**
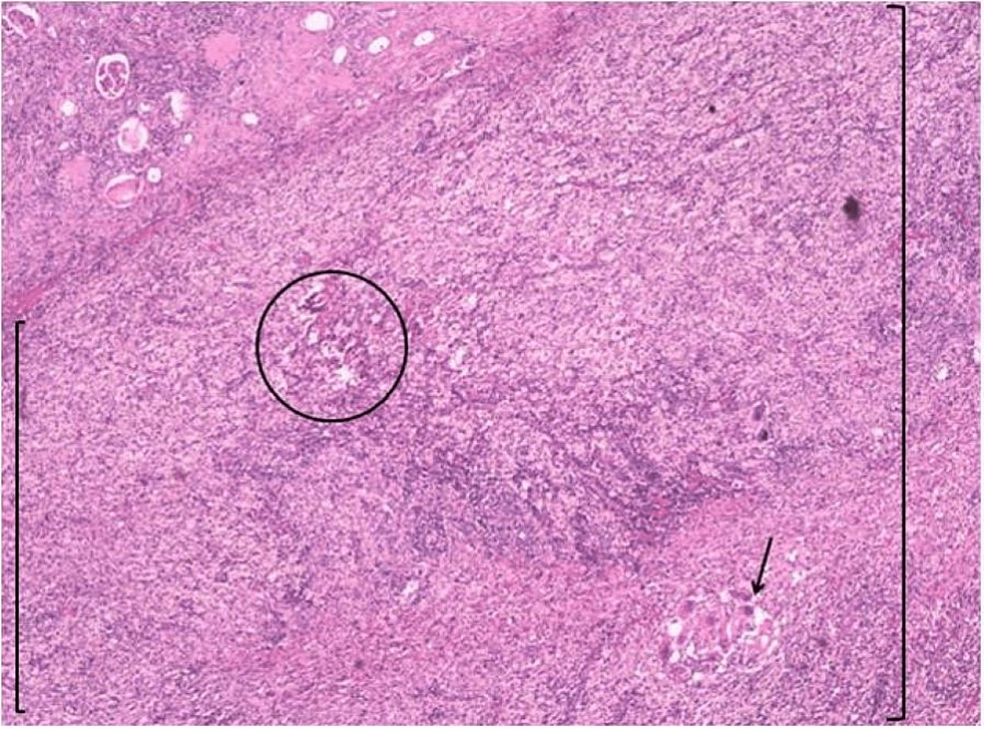
H&E stain showing a xanthogranulomatous inflammatory reaction (brackets) with foamy macrophages (circle) and a granuloma with giant mononuclear cells (arrow) H&E: hematoxylin and eosin

Figure 1 shows thoracic and abdominal computed tomography (CT) with 1) a structural alteration of the left kidney, replaced by multiple cysts, some of which with calcium content, involving the perirenal space and the posterior wall of the stomach (Figure 1A and Figure 1C); 2) bulky, necrotic, left latero-aortic conglomerate of 9x2.6x2.2 centimeters with compression of the left renal vein, and 3) left pleural effusion (Figure 1B).

A diagnostic and therapeutic thoracentesis and an ultrasound-guided drainage of one of the largest cysts were performed. The pleural effusion was purulent and biochemical analysis revealed pH 7, 207.080 leucocytes with polymorphonuclear predominance, LDH 8399 UI/L, glucose 5 mg/dL, and total protein 4.2 g/dL. The gross appearance of the content of the cyst was also purulent with microbiological identification of Escherichia coli resistant to amoxicillin/clavulanate on both. A fistula between perirenal and pleural spaces was suspected, justifying the presence of a typical urinary microorganism in the pleural space. A drain was placed in the left pleural cavity and left kidney, and she was started on ceftriaxone 2 grams daily. Cytological examination of the pleural effusion and cyst did not find malignant cells.

**Figure 3:**
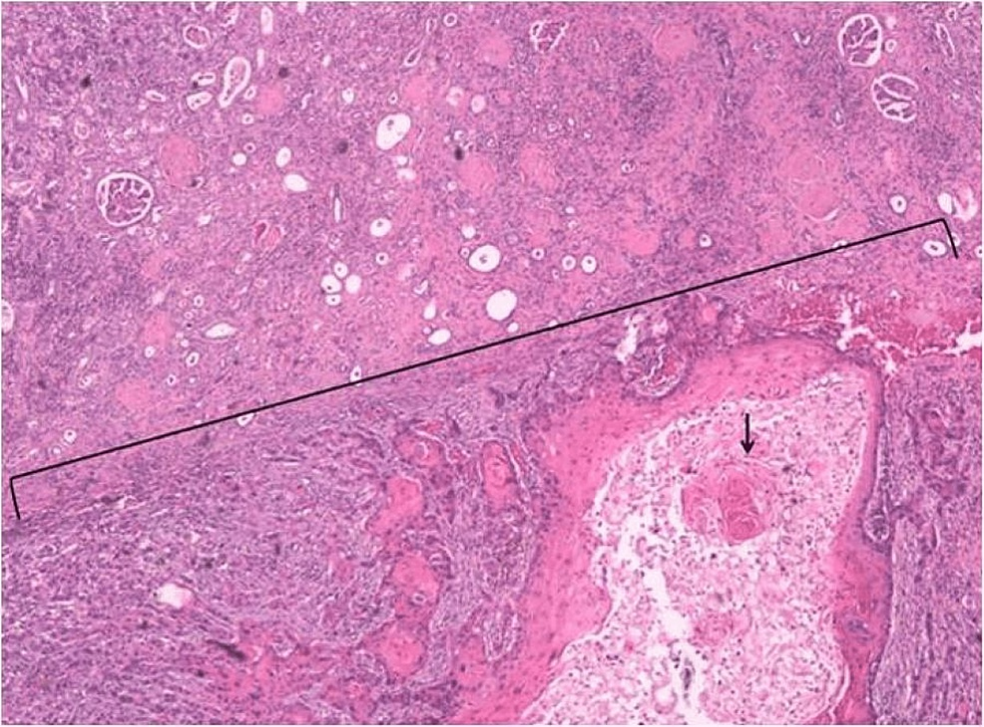
H&E stain revealing a moderate to poorly differentiated primary squamous cell carcinoma infiltrating the renal parenchyma (brackets), with keratin pearls (arrow) H&E: hematoxylin and eosin

After 10 days of antibiotic therapy, the patient underwent total left nephrectomy by lumbotomy, which was laborious due to the adherence of the kidney to adjacent structures. The clinical evolution in the immediate postoperative period was favorable, with defervescence and a decrease in inflammatory markers. However, the patient subsequently presented rapid worsening of constitutional symptoms (anorexia, continuous weight loss, and fever without response to antibiotic therapy despite no additional microbiological findings) and progressive ascites, previously absent. Two weeks after surgery, she was totally confined to bed. At this time, a histological examination of the kidney showed xanthogranulomatous pyelonephritis and a moderate to poorly differentiated, multifocal, and keratinizing primary squamous cell carcinoma of the renal pelvis (Figures 2, 3). The tumor invaded the ureter, renal parenchyma, adipose tissue of the renal sinus, and perirenal adipose tissue, with no lymph nodes identified in the surgical specimen.

**Figure 4:**
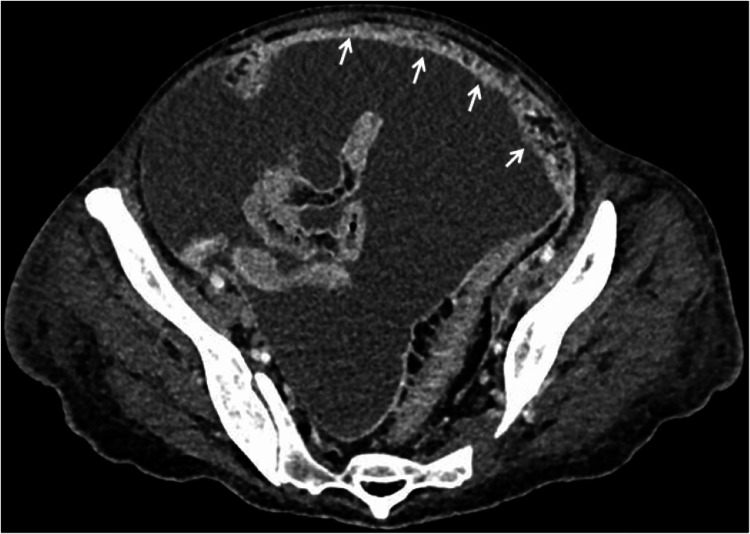
Abdominal and pelvic CT revealing tense ascites compressing the colon and aspects suggesting peritoneal carcinomatosis with an anterior epiploic “omental-cake” pattern (arrows)

For further clarification, abdominal and pelvic CT was repeated showing significant ascites and peritoneal carcinomatosis with an anterior epiploic “omental-cake” expression (Figure 4).

Considering a stage IV neoplasia and an Eastern Cooperative Oncology Group (ECOG) performance status of 4, the patient did not begin chemotherapy, and the best supportive and palliative care was provided. With corticosteroids, loop diuretics, and antisecretory agents, effective symptomatic control was achieved. She died in the fourth week of hospitalization.

## Discussion

XPN is a rare form of chronic pyelonephritis. Most cases occur in settings of obstruction due to infected renal stones and probably represent an abnormal immune response to bacterial infection [1]. The affected kidney usually manifests as a nonfunctioning hydronephrotic kidney, in which histopathological examination reveals a massive destruction of renal parenchyma that is replaced by foamy, lipid-laden macrophages [5].

Clinical presentation is usually subacute with abdominal or flank pain and fever being the most frequent complaints, sometimes lasting months. Constitutional symptoms, including malaise, anorexia, and weight loss, can also be found, especially in those with a more insidious disease course. Chuang et al. (1992) conducted a retrospective study including 36 patients with XPN to analyze clinical symptoms and signs at presentation, laboratory and radiographic findings, and the result of urine and blood cultures. The most frequent clinical symptoms and signs were flank/abdominal pain (75%), followed by fever (42%), palpable mass (11%), and weight loss (6%) [5]. With the exception of a palpable mass, our patient had all the remaining signs and symptoms reported. In the same study, the etiology of XPN was also evaluated, with urinary obstruction in the context of urolithiasis being the most frequent (83%), including renal calculi (42%), staghorn stone (31%) and ureteral calculi (19%) [5]. Unilateral obstruction of the urinary tract of other etiologies (secondary ureteropelvic junction stricture and ureteral tumor) was responsible for the other cases [5]. Of 33 cultures, 27 were positive (82%), approximately one-third with mixed infections. Escherichia coli was identified in 67% of the cultures, Proteus mirabilis in 26%, Staphylococcus species in 19%, and Streptococcus species in 7% while Pseudomonas and Enterococcus species were much rarer. These findings are concordant with other reports in the literature, with Escherichia coli and Proteus mirabilis being the most frequent microorganisms associated with this rare entity [1,6].

Extension to other organs such as the intestine, spleen, skin, or lung is rare, but the process may lead to fistula formation [1], as seen in our case, affecting other organs and ending up in complex surgical intervention. In Chuang et al.'s retrospective study, there were two patients (5,6%) whose initial clinical presentation with bloody stool and cutaneous purulent discharge was a consequence of a fistula to the descending colon and skin, respectively [5]. Fistula formation is, therefore, an infrequent complication of a disease that is rare in itself.

Since XPN is associated with complete destruction of the kidney, its treatment is surgical and almost always involves total nephrectomy and closure of any existing fistulas. It is important to ensure an initial course of antibiotics to control local infection. In the absence of complications, surgery is curative.

The most important differential diagnosis is renal carcinoma since its clinical presentation and imaging appearance can be similar. Other conditions important to distinguish histologically from XPN are renal parenchymal malakoplakia and megalocytic interstitial nephritis [7].

In contrast with urothelial carcinomas, primary squamous cell carcinoma of the renal pelvis and ureter are very rare and represent less than 1% of all urinary tract neoplasms [2,3], with women being affected more frequently than men [4]. Perez-Montiel et al. (2007) in a study including 108 patients with high-grade urothelial carcinoma of the renal pelvis noted squamous differentiation in 14 cases and SCC in only one case [8].

These rare malignancies can be associated with renal stone disease [3,4], probably as a consequence of chronic inflammation or infection due to urinary stasis, which can lead to squamous metaplasia of the urothelium with subsequent carcinomatous transformation. Abuse of analgesics containing phenacetin is also a risk factor for renal SCC [3,4,9,10]. Due to its rarity, most of the current knowledge about XPN and associated SCC of the renal pelvis is based on small case series or isolated case reports (Table 2) [4,9,11-19].

**Table 2:** Case reports concerning concomitant XPN and renal pelvis SCC N/D - no data; XPN - xanthogranulomatous pyelonephritis; SCC - squamous cell carcinoma All patients presented hydronephrosis and a non-functioning kidney. The two deaths not related to SCC corresponded to an acute myocardial infarction. * Complicated by a renohepatic fistula ** Complicated by a pyelogastric fistula

	Age	Sex	Clinical presentation	Duration of symptoms	Renal stone	Previous stone surgery	Surgery	Outcome
Mardi et al., 2010 [4]	65	Male	Lower back pain, fever	2 months	Renal calculi	No	Left nephrectomy	N/D
	65	Female	Flank pain	N/D	Renal calculi	No	Right nephrectomy	N/D
Hippargi et al., 2016 [9]	61	Male	Flank pain, dysuria	6 months	Renal calculi	Yes	Right nephrectomy	N/D
Hosseinzadeh et al., 2020 [11]	59	Female	Flank pain, hematuria	3 months	Staghorn	No	Right nephrectomy	Death in 1 year due to disease progression
Jain et al., 2011 [12]	50	Male	Flank pain, hematuria	2 months	Staghorn	No	Right nephrectomy	N/D
	87	Male	Flank pain	2 months	Staghorn	Yes	Left nephrectomy	Inpatient death not related to SCC
	50	Male	Flank pain	3 months	Renal calculi	No	Left nephrectomy	Alive at 3 months in a cypslatin-based regimen
	53	Male	Flank pain	5 months	Renal calculi	Yes	Right nephrectomy	Alive at 5 months in a cypslatin-based regimen
Nachiappan et al., 2016 [13]	60	Female	Flank pain, fever	3 months	Staghorn	No	Left nephrectomy	N/D
Varshney et al., 2023 [14]	66	Male	Flank pain	20 days	Staghorn	Yes	Right nephrectomy	Death in 6 months not related to SCC*
Kanodia et al., 2015 [15]	60	Male	Flank pain	N/D	Renal calculi	No	Left nephrectomy	N/D
Yousof et al., 2014 [16]	45	Male	Hip pain	6 months	Staghorn	No	Left nephrectomy	N/D
Chang et al., 2021 [17]	69	Female	Flank pain	Several days	Staghorn	No	Right nephrectomy	Alive at 4 months without adjuvant therapy
Kasahara et al., 2020 [18]	70	Female	Fatigue, fever	> 1 month	Staghorn	No	Left nephrectomy	Death in 2 months due to disease progression
Veerabathini et al., 2020 [19]	54	Male	Fatigue, anorexia, weight loss	1 month	Staghorn	No	Palliative gastrojejunostomy	Inpatient death due to disease progression**

Renal pelvis SCC may be first suspected in patients presenting with flank pain and urolithiasis with radiological evidence of hydronephrosis and a non-functioning kidney. Since other rare entities, such as XPN, may have similar presentations, the definitive diagnosis implies a careful assessment of the surgical specimen and examination of histopathological sections to avoid misdiagnosing this disease and allow early diagnosis [20]. In patients undergoing treatment for renal calculi, whenever possible, especially in those with staghorn calculi, biopsy from the renal pelvis or calyceal wall should be considered due to the increased susceptibility to malignancy [15]. In our patient, if a close follow-up of her known nephrolithiasis had been carried out, she could have performed a biopsy, allowing for an earlier diagnosis of both XPN and SCC and avoiding a fatal outcome.

Its prognosis is poor because it usually presents in advanced stages and lacks the characteristic presentation of common renal cell carcinoma [3,9]. In 2007, Holmang et al., in a study including 808 patients, found that only 4% of 65 patients with SCC had stage pTa/pT1/pT2 tumors compared to 62% of 743 with urothelial carcinoma [10]. They also reported that five-year survival was less than 10% and the average postoperative survival rate did not exceed seven months.

Regarding treatment of this neoplasia, surgery is recommended even in metastatic disease as long as the patient is fit for the procedure [3,9,20]. Once surgical dissection is difficult during nephrectomy for complicated nephrolithiasis, in a nonfunctional kidney, the possibility of concomitant neoplasia should be kept in mind, and it is advisable to dissect in a plane outside Gerota’s fascia as for radical nephrectomy [4]. Adjuvant and neoadjuvant chemotherapy have only a marginal effect on treatment [3,10], contributing to the poor prognosis of this neoplasia and highlighting the need for adequate clinical and imaging surveillance in patients with known renal lithiasis or other risk factors to avoid progression to SCC of the renal pelvis.

The rarity of concomitant XPN and SCC of the renal pelvis is responsible for the main limitation of the studies and case reports concerning the treatment of this disease. Since there are few reported cases of SCC, little is known about the best treatment for metastatic cases [3]. 

## Conclusions

Although XPN and SCC of renal pelvis are rare entities, the association between both is well-established, with XPN constituting a risk factor for the development of SCC. In a patient presenting with complicated nephrolithiasis, hydronephrosis, and a non-functioning kidney, the suspicion of possible concomitant SCC of the renal pelvis must be kept in mind due to its therapeutic and prognostic implications, distinct from those of XPN alone.

Since the prognosis of renal SCC is poor, a prospective study comparing different therapeutic approaches would be useful, but the scarcity of this disease makes it difficult to perform.
